# Impact of maternal HIV infection on pregnancy outcomes in southwestern China – a hospital registry based study

**DOI:** 10.1017/S0950268818003345

**Published:** 2019-03-08

**Authors:** M. Yang, Y. Wang, Y. Chen, Y. Zhou, Q. Jiang

**Affiliations:** 1 Department of HIV/AIDs and STIs Control, Xuhui Center for Disease Control and Prevention, Shanghai, China; 2 School of Epidemiology, Public Health and Preventive Medicine, Faculty of Medicine, University of Ottawa, Ottawa, Ontario, Canada; 3 Department of Epidemiology, School of Public Health, Fudan University, Shanghai, China; 4 Key Laboratory of Public Health Safety, Ministry of Education, Fudan University, Shanghai, China

**Keywords:** Adverse pregnancy outcomes, HIV/AIDS, LBW, low Apgar score, PTD

## Abstract

Globally, human immune deficiency virus (HIV)/acquired immune deficiency syndrome (AIDS) continues to be a major public health issue. With improved survival, the number of people living with HIV/AIDS is increasing, with over 2 million among pregnant women. Investigating adverse pregnant outcomes of HIV-infected population and associated factors are of great importance to maternal and infant health. A cross-sectional data collected from hospital delivery records of 4397 mother–infant pairs in southwestern China were analysed. Adverse pregnant outcomes (including low birthweight/preterm delivery/low Apgar score) and maternal HIV status and other characteristics were measured. Two hundred thirteen (4.9%) mothers were HIV positive; maternal HIV infection, rural residence and pregnancy history were associated with all three indicators of adverse pregnancy outcomes. This research suggested that maternal population have high prevalence in HIV infection in this region. HIV-infected women had higher risks of experiencing adverse pregnancy outcomes. Rural residence predisposes adverse pregnancy outcomes. Findings of this study suggest social and medical support for maternal-infant care needed in this region, selectively towards rural areas and HIV-positive mothers.

## Introduction

Although lives of human immune deficiency virus (HIV)-infected population are prolonged tremendously due to highly active antiretroviral therapy, HIV infection continues to be a major public health issue across the globe [1]. By 2017, there were approximately 1.1 million HIV-infected pregnant women [2]. As a special population, HIV-infected pregnant women can transmit HIV vertically to their children during pregnancy, delivery or breastfeeding, with an overall transmission rate between 15% and 45%, if without any intervention [2]. And mother-to-child transmission (MTCT) of HIV is the major route of acquiring HIV for children <15 years of age. Researches have also indicated that HIV infection during pregnancy can have severe impact on the foetus, including prematurity, low birthweight (LBW) and low Apgar score [3–5], as well as neurodevelopmental problems [6].

LBW and prematurity are important contributors for perinatal morbidity and mortality [7–9]. Studies have reported associations of LBW/prematurity with inhabited growth, delay in cognitive development and even chronic disease later in life [10–12]. To date, inconsistent results have been reported regarding the association between maternal HIV infection and adverse pregnancy outcomes (including LBW, prematurity and low Apgar score). Some studies indicated that maternal HIV infection could increase the risk of LBW and prematurity [3–5], while others reported no significant association between maternal HIV status and LBW/prematurity [13, 14].

China has been a country of low HIV prevalence overall, but some southwestern provinces are experiencing endemics of HIV/acquired immune deficiency syndrome (AIDS), including Sichuan and Yunnan [15]. Data from some surveillance sites showed a prevalence of 1% or more in some endemic areas [15], and therefore, a high prevalence of HIV infection among pregnant women is anticipated in these areas [16]. However, current research on pregnancy outcomes of HIV-infected women in China is limited and results may not be generalisable to a broader population due to small sample size [17]. This cross-sectional study collected data from a high-risk county of southwestern China, to explore the prevalence of HIV-infection among pregnant women, and its association with pregnancy outcomes.

## Methods

### Study sites and participants

The study was conducted in a county of a Yi Autonomous Prefecture, southwestern China, where ethnic Yi (one of Chinese major ethnic minority group) reside predominately. Geographically, this prefecture serves as an important production and distribution centre for drug smuggling from ‘Golden Triangle’ area, the percentage of local residents using injection drugs or involved in drug trafficking are high [18, 19]. Culturally, Yi people are open concerning sex. Casual sexual behaviours without protection are quite common within this community [19, 20]. Many young people especially male often engage in risky sexual practices [21]. Since the first HIV case was identified in this prefecture in 1995, it has been one of the most endemic areas for HIV infection in China [22]. By the end of 2013, there were over 25 000 reported cases of HIV infection in this prefecture alone, accounting for 50% of all reported cases in Sichuan province [19, 23].

In the county, there were only three hospitals (People's Hospital, county's Maternal and Child Health Care Hospital (MCH) and a township hospital) that have maternal-child care services during the study period, where pregnant women can deliver babies. For every pregnant woman who admits into these hospitals for delivery, information for both mother and infant was recorded by healthcare professionals.

Regarding HIV prevention and control efforts, there are 18 CDC centres within this prefecture and one located in this county. Free HIV test service is provided for every visitor. For pregnant women, HIV test is also provided freely along with other tests for every women seeking antenatal care services in local hospitals. Despite the above services provided, majority of the pregnant women do not seek antenatal care services before delivery due to complicated reasons (e.g. poor public transportation, limited education and awareness for antenatal care, economic reasons).

### Data collection

Due to better medical resources, majority of pregnant women give births in the People's Hospital or the county's MCH, and very few of them give births in the township hospital. For this reason, we only collected hospital delivery data from the county's MCH and People's Hospital.

A total of 4933 delivery data of the county's MCH and People's Hospital from June 2014 to March 2015 were retrieved. Data were entered into EpiData by trained investigators, and were de-identified by removing participants’ names and ID number to protect privacy. Among them, 142 premature deaths (including 114 perinatal deaths, 25 stillbirths and three neonatal deaths) and 143 non-singleton births were excluded. In total, 4397 delivery data were analysed after 257 were excluded due to missing data on maternal HIV information. This study was approved by the Ethical Review Committee of Fudan University, Shanghai, China.

### Statistical analysis

#### Maternal characteristics

Maternal age, ethnicity, place of residence, hospital of delivery, HIV status, syphilis status, pregnancy history, delivery history and mode of delivery were included in the analysis. Maternal age was further dichotomised as <35 years and ⩾35 years. Place of residence was defined as urban (yes, no). Delivery hospital was classified as People's hospital and county's MCH. Ethnicity was categorised into three groups as Han (Chinese predominant ethnic group), Yi (one of the Chinese major ethnic minority groups) and other. Pregnancy history was dichotomised as 1 and ⩾2. Mode of delivery was categorised as vaginal delivery, caesarean section (C-section) with medical indication and C-section without medical indication.

#### Infant characteristics (pregnancy outcomes)

Birthweight, gestational weeks and Apgar scores (a summary of appearance, pulse, grimace, activity, respiration) at 1, 5 and 10 min were selected for analysis. LBW was defined as <2500 g (no, yes) [24]. Number of gestational weeks was categorised into three groups as <34 weeks, 34–36 weeks and ⩾37 weeks, of which gestation of <34 weeks and 34–36 weeks were defined as a preterm delivery (PTD). For Apgar scores at 1, 5 and 10 min, low Apgar score was all defined as <7 (no, yes). An infant with Apgar score <7 indicates that the newborn may need medical attention [25].

Data were analysed using SAS 9.4 for windows (SAS Institute, Inc., Cary, NC). Individual characteristics by maternal HIV status were presented using descriptive statistics. *χ*^2^ test of independence or Fisher's exact test was used to assess the statistical significance of associations between maternal HIV status and maternal/infant characteristics where appropriate. Logistic regression analysis was used to explore the potential risk factors for adverse pregnancy outcomes. We conducted three independent sets of logistic regression analysis for LBW, PTD and low Apgar score at 10 min. For each set of logistic analysis, univariate analyses were performed first, followed by multivariate analyses using a backward elimination approach with all potential confounders entering the model. Missing value of three independent variables (urban residency with 20.9% missing, maternal syphilis status with 26.9% missing and delivery hospital with 27.5% missing) were replaced by multiple imputation (fully conditional specification method, 50 imputations). Odds ratios (ORs) and 95% confidence interval (95% CIs) were calculated. Statistical significance was set at *P* value of <0.05.

## Results

### Maternal and infant characteristics

#### Maternal characteristics

Among the 4379 mother–infant pairs, 213 (4.9%) mothers were HIV positive; 4152 (95.8%) were ethnic Yi and 178 (4.1%) were ethnic Han; with a mean age of 27.6 years (s.d. = 6.6); majority had two or more pregnancy history (59.8%) and previous delivery history (83.3%) (Table 1). Compared with mothers who were HIV negative, mothers who were HIV positive differed significantly in ethnicity (*P* = 0.022), pregnancy history (*P* = 0.009), gestational duration (*P* = 0.016), syphilis status (<0.0001), urban residency (*P* = 0.004) and delivery hospital (*P* = 0.0008) (Table 1).

**Table 1.** Characteristics of mother and infant pairs by maternal HIV status.

Characteristics	HIV positive (*n* = 213)	HIV negative (*n* = 4166)	Total (*N* = 4379)	*P*
	*n* (%)	*n* (%)	*N* (%)	
*Maternal characteristics*				
Age				
<35 years	169 (79.7)	3474 (83.8)	3643 (83.6)	0.128
⩾35 years	43 (20.3)	671 (16.2)	714 (16.4)	
Mean (± s.d.)	27.6 ± 6.6			
Race				
Yi	208 (99.5)	3944 (95.6)	4152 (95.8)	**0.022**
Han	1 (0.5)	177 (4.3)	178 (4.1)	
Other	0 (0.0)	6 (0.1)	6 (0.1)	
Gravidity (times)				
1	66 (31.6)	1680 (40.7)	1746 (40.2)	**0.009**
⩾2	143 (68.4)	2452 (59.3)	2595 (59.8)	
Parity (times)				
0	29 (13.9)	697 (16.9)	726 (16.7)	0.260
⩾1	180 (86.1)	3438 (83.1)	3618 (83.3)	
Mode of delivery				
Vaginal delivery	169 (80.1)	3162 (76.5)	3331 (76.7)	0.217
C-section (with medical indication)	38 (18.0)	925 (22.4)	963 (22.2)	
C-section (without medical indication)	4 (1.9)	47 (1.1)	51 (1.2)	
PTD				
<34 weeks	20 (9.6)	203 (4.9)	223 (5.1)	**0.0057**
34–36 weeks	0 (0.0)	29 (0.7)	29 (0.7)	
⩾37 weeks	188 (90.4)	3900 (94.4)	4088 (94.1)	
Syphilis				
Negative	143 (92.9)	2986 (98.0)	3129 (97.8)	**<0.0001**
Positive	11 (7.1)	61 (2.0)	72 (2.2)	
Urban				
No	173 (97.7)	3012 (91.7)	3185 (92.0)	**0.004**
Yes	4 (2.3)	273 (8.3)	277 (8.0)	
Delivery hospital				
People's hospital	106 (69.3)	2482 (80.4)	2534 (79.9)	**0.0008**
MCH	47 (30.7)	592 (19.6)	639 (20.1)	
*Infant characteristics*				
LBW (g)				
<2500	33 (15.6)	342 (8.3)	375 (8.6)	**0.0002**
⩾2500	178 (84.4)	3789 (91.7)	3967 (91.4)	
Birth defect				
No	209 (99.5)	4130 (99.6)	4339 (99.6)	0.569
Yes	1 (0.5)	16 (0.4)	17 (0.4)	
Apgar score 1				
<7	3 (1.5)	38 (0.9)	41 (1.0)	0.445
⩾7	204 (98.5)	4085 (99.1)	4289 (99.0)	
Apgar score 5				
<7	10 (4.8)	88 (2.1)	98 (2.3)	**0.026**
⩾7	197 (95.2)	4035 (97.9)	4232 (97.7)	
Apgar score 10				
<7	27 (13.0)	284 (6.9)	311 (7.2)	**0.0008**
⩾7	180 (87.0)	3839 (93.1)	4019 (92.8)	

Bold values are *p*<0.05.

Numbers not adding up due to missing; MCH, Maternal and Child Health Care Hospital.

#### Infant characteristics

Among infants, 252 (5.8%) were born before 37 weeks of gestation (including 223 before 34 weeks and 29 within 34–36 weeks of gestation) and 375 (8.6%) weighed <2500 g (Table 1). Birth defects were reported in 17 (0.4%) infants (Table 1). Infants born to HIV-positive mothers differed significantly in LBW (*P* < 0.0002) and low Apgar score recorded at 5 and 10 min (*P* = 0.026 and *P* = 0.0008, respectively) as compared with infants born to HIV negative mothers (Table 1).

### Pregnancy outcomes

#### LBW

Results from the multivariate analysis revealed four predictors associated with LBW (Table 2). HIV positive (OR 1.93, 95% CI 1.27–2.94), rural residency (OR 2.44, 95% CI 1.06–5.66) and having PTD (OR_<34 weeks_ 7.60, 95% CI 5.62–10.29; OR_34–36 weeks_ 15.04, 95% CI 7.11–31.83) were positively and independently associated with LBW (Table 2). However, previous pregnancy history (OR 0.74, 95% CI 0.59–0.92) was negatively associated with LBW (Table 2). Syphilis, as a typical sexually transmitted infection, was associated with LBW in bivariate analysis, however, the association did not reach statistical significance in multivariate analysis.

**Table 2.** Prevalence and correlates of LBW among sample mother–infant pairs.

Variables	LBW	Univariate		Multivariate	
	(%, *n*/*N*)	OR (95% CI)	*P*	aOR (95% CI)	*P*
Age(years)					
<35	8.8 (319/3611)	1			
⩾35	7.7 (55/709)	0.87 (0.64–1.17)	0.352		
Race					
Han	3.4 (6/177)	1			
Yi	8.8 (363/4116)	2.75 (1.21–6.26)	**0.016**		
Other	0 (0/6)	Null			
Urban					
Yes	6.5 (18/276)	1		1	
No	9.0 (284/3153)	1.42 (0.87–2.32)	0.165	2.44 (1.06–5.66)	**0**.**036**
Delivery hospital					
People's hospital	9.6 (240/2507)	1			
MCH	6.6 (42/634)	0.67 (0.48–0.94)	**0.021**		
HIV status					
Negative	8.3 (342/4131)	1		1	
Positive	15.6 (33/211)	2.05 (1.39–3.03)	**0.0003**	1.93 (1.27–2.94)	**0.002**
Syphilis					
Negative	8.9 (276/3098)	1			
Positive	16.9 (12/71)	2.08 (1.11–3.92)	**0.023**		
Gravidity					
1	10.3 (178/1733)	1		1	
>1	7.5 (192/2574)	0.70 (0.57–0.87)	**0.001**	0.73 (0.59–0.92)	**0.007**
Parity					
None	11.5 (83/722)	1			
⩾1	8.0 (288/3587)	0.67 (0.52–0.87)	**0.003**		
Mode of delivery					
Vaginal delivery	9.0 (298/3321)	1			
C-section (with medical indication)	7.8 (75/962)	0.86 (0.66–1.12)	0.255		
C-section (without medical indication)	0 (0/51)	<0.001 (<0.001–>999.99)	0.968		
PTD					
<34 weeks	37.4 (83/222)	8.31 (6.16–11.20)		7.60 (5.62–10.29)	**<0.0001**
34–36 weeks	55.6 (15/27)	17.37 (8.05–37.48)	**<0.0001**	15.04 (7.11–31.83)	**<0.0001**
⩾37 weeks	6.7 (272/4056)	1		1	

Bold values are *p*<0.05.

Numbers not adding up due to missing; MCH, Maternal and Child Health Care Hospital.

#### PTD

Results from multivariate analysis indicated four predictors associated with PTD (Table 3). Mothers who were HIV positive (OR 1.76, 95% CI 1.06–2.91) and lived in rural areas (OR 2.11, 95% CI 1.09–4.08) were positively and independently associated with PTD, while delivery in county's MCH (OR 0.43, 95% CI 0.23–0.79) and had previous pregnancy history (OR 0.74, 95% CI 0.56–0.97) were negatively associated with PTD (Table 3).

**Table 3.** Prevalence and correlates of PTD among sample mother–infant pairs

Variables	Preterm birth	Univariate		Multivariate	
	(%, *n*/*N*)	OR (95% CI)	*P*	aOR (95% CI)	*P*
Age(years)					
<35	5.9 (212/3608)	1			
⩾35	5.4 (38/710)	0.91 (0.64–1.29)	0.585		
Race					
Han	4.0 (7/177)	1			
Yi	5.9 (244/4119)	1.53 (0.71–3.29)	0.278		
Other	0.0 (0/6)	Null			
Urban					
Yes	3.6 (10/277)	1		1	
No	6.7 (211/3172)	1.90 (1.00–3.63)	0.051	2.11 (1.09–4.08)	**0**.**026**
Delivery hospital					
People's hospital	4.6 (117/2534)	1		1	
MCH	2.2 (14/630)	0.47 (0.27–0.82)	**0.008**	0.43 (0.23–0.79)	**0.006**
HIV status					
Negative	5.6 (232/4132)	1		1	
Positive	9.6 (20/208)	1.79 (1.11–2.89)	**0.018**	1.76 (1.06–2.91)	**0.029**
Syphilis					
Negative	4.1 (128/3120)	1			
Positive	8.3 (6/72)	2.13 (0.91–4.99)	0.084		
Gravidity					
1	6.9 (119/1731)	1		1	
>1	5.2 (133/2575)	0.74 (0.57–0.95)	**0.020**	0.74 (0.56–0.97)	**0.027**
Parity					
None	8.2 (59/724)	1			
⩾1	5.4 (193/3584)	0.64 (0.47–0.87)	**<0.004**		
Mode of delivery					
Vaginal delivery	5.6 (185/3300)	1			
C-section (with medical indication)	6.7 (64/957)	1.21 (0.90–1.62)	0.210		
C-section (without medical indication)	2.0 (1/51)	0.34 (0.05–2.45)	0.283		

Bold values are *p*<0.05.

Numbers not adding up due to missing; MCH, Maternal and Child Health Care Hospital.

#### Low Apgar score

The multivariate analysis revealed six predictors associated with low Apgar score at 10 min. Mothers who were HIV positive (OR 2.07, 95% CI 1.34–3.02), 35 years or older (OR 1.70, 95% CI 1.24–2.32), from ethnic Yi (OR 2.69, 95% CI 1.05–6.89), lived in rural areas (OR 2.02, 95% CI 1.07–3.81) and had C-section deliveries under medical indication (OR 2.74, 95% CI 2.12–3.53) were more likely to have an Apgar score of <7. Similar to LBW and PTD, previous pregnancy history (OR 0.64, 95% CI 0.50–0.83) was negatively associated with low Apgar score at 10 min (Table 4).

**Table 4.** Prevalence and correlates of low Apgar score (<7) at 10 min among newborns of the study sample

Variables	Apgar score <7 at 10 min	Univariate		Multivariate	
	(%, *n*/*N*)	OR (95% CI)	*P*	aOR (95% CI)	*P*
Age(years)					
<35	6.6 (239/3602)	1		1	
⩾35	9.8 (69/706)	1.52 (1.15–2.02)	**0**.**003**	1.70 (1.24–2.32)	**0**.**0009**
Race					
Han	2.8 (5/178)	1		1	
Yi	7.4 (302/4103)	2.75 (1.12–6.73)	**0.027**	2.69 (1.05–6.89)	**0.039**
Other	16.7 (1/6)	Null			
Urban					
Yes	4.0 (11/276)	1		1	
No	8.2 (256/3140)	2.14 (1.15–3.96)	**0.016**	2.02 (1.07–3.81)	**0.030**
Delivery hospital					
People's hospital	8.7 (218/2504)	1			
MCH	5.5 (34/621)	0.61 (0.42–0.88)	**0.009**		
HIV status					
Negative	6.9 (284/4123)	1		1	
Positive	13.0 (27/207)	2.03 (1.33–3.09)	**0.001**	2.07 (1.34–3.20)	**0.001**
Syphilis					
Negative	8.0 (245/3082)	1			
Positive	12.7 (9/71)	1.68 (0.83–3.42)	0.152		
Gravidity					
1	8.3 (144/1782)	1		1	
>1	6.4 (164/2566)	0.75 (0.60–0.95)	**0.016**	0.64 (0.50–0.83)	**0.0008**
Parity					
None	8.3 (60/721)	1			
⩾1	6.9 (248/3576)	0.82 (0.61–1.1)	0.188		
Mode of delivery					
Vaginal delivery	5.4 (179/3306)	1		1	
C-section (with medical indication)	13.2 (127/962)	2.66 (2.09–3.38)	**<0.0001**	2.74 (2.12–3.53)	**<0.0001**
C-section (without medical indication)	5.9 (3/51)	1.09 (0.34–3.54)	0.884	1.16 (0.35–3.88)	0.805

Bold values are *p*<0.05.

Numbers not adding up due to missing; MCH, Maternal and Child Health Care Hospital.

## Discussion

In this study, we estimated the prevalence of HIV infection (4.9%) and associated adverse pregnancy outcomes among pregnant women attending two local hospitals in a county of a Yi Autonomous Prefecture, southwestern China. HIV-infected women had higher risks of experiencing adverse pregnancy outcomes, including LBW, PTD and low Apgar score. Rural residency had negative effects on pregnancy outcomes, while previous pregnancy history seems prevent pregnant women from experiencing adverse pregnancy outcomes. In addition, advanced maternal age and being ethnic Yi have negative impact on rating of newborn's Apgar score.

Majority of HIV-positive women did not know their HIV status before admission. This reflected the under-utilisation of antenatal care service in this area. In our study, 92% pregnant women were from rural areas of this prefecture. Poor public transportation might be a major reason for not utilising such services. In addition, women locally generally have limited education, many of them don't speak mandarin. According to recent literature, there is still a certain percentage of pregnant women who still deliver at home even today [26]. Despite economic and other social complications, this might be another reason antenatal care service were under-utilised in this area, resulting delayed HIV testing among pregnant women.

The prevalence of HIV infection was much higher compared with other HIV endemic provinces (0.5% in Yunnan and 0.2% in Xinjiang [27, 28]) in China. This may partly due to the fact that Yi people are more likely to engage in risky sexual practices. The percentage of young people engage in casual sex is high. Study have reported a percentage of over 50% in some social gatherings among ethnic Yi, such as attending a wedding or going to a township fair [29]. In addition, culturally, young men from ethnic Yi are proud of having multiple sex partners and widows usually remarry with brothers of her husband to maintain his line [21]. As a major rout of HIV transmission, these high risk sexual conducts can increase this risk, especially when condom use is infrequent and access to it is limited [23, 20]. Located in a remote mountain with harsh climate, the Yi autonomous prefecture is poorly developed. Deprived public transportation and limited medical resources restricted local people from getting medical services or health care information in time, which in return increased the risk of HIV transmission. In addition, local Yi people are generally less educated and only a small percentage of them can speak mandarin [20], which makes health education less informative. The combined effects of all these factors made the current situation of HIV/AIDS even worse in these areas. As regard to syphilis (another common sexual transmitted disease), an overall prevalence of 2.2% in this community is also much higher than its most counterparts from other regions of China. This prevalence is even higher than some high risk groups, such as female sex workers. Data from national HIV surveillance sites among female sex workers had only revealed a prevalence of syphilis around 1% in different years [30]. We think the two phenomena are similar in this region, with many complicated reasons, including risky sexual practices due to its unique culture and other social reasons, its physical location as ‘Golden Triangle’ area for drug smuggling and overall less development (poor economic, deprived public transportation and limited education). As generally known, drug use and risky sexual practices can have synergistic effect towards sexually transmitted infections (STIs), including HIV and syphilis. We think this is a large part that accounted for syphilis as well as HIV infection in this region. Although we do not deny the above reasons, China as a whole has been experiencing a dramatic resurgence in syphilis over the past 20 years due to its rapid social changes. With this sufficient ‘substrate’, those above ‘catalysts’ we discussed would definitely boosted the ‘chemical reaction’, leading a great amount of syphilis/HIV infection in this region. To thwart HIV and syphilis co-epidemic in this region, a strong government commitment is essential. From the clinical aspect, medical resource from outside need to be directed to this undeveloped community, with substantial efforts placed on village level health stations, such as free HIV/syphilis screening services. From the public health aspect, health education to promote safe sex conduct and distributing condom might be helpful. Antenatal health care service might be the last door guardian in such circumstance.

The prevalence of LBW among HIV negative mothers was 8.3%, while it was 15.6% for HIV-positive mothers. Consistent with previous results [31–33], maternal HIV infection was independently associated with LBW, with a 1.9-fold increased risk in the current study. Altered immune responses of mother and infant pairs are likely an important reason for this association [34–36]. Studies have demonstrated a possible mechanism that maternal HIV infection can lead to an increase of proinflammatory cytokine on maternal placental cells, which is an indicator of immune response towards infection. This change may interrupt the normal build-up of an infant's immune system, which in return can interfere with a normal development of the foetus, causing MTCT of HIV and other adverse pregnancy outcomes. Studies have also indicated that women are immunocompromised when pregnant [37], which makes them more vulnerable towards reproductive tract infections that can contribute to adverse pregnancy outcomes.

A LBW prevalence of 8.3% among HIV negative mothers was significantly low as compared with HIV-positive mothers, but was nearly twice compared with general population of China [38]. The high prevalence may be due to, at least in part, the disadvantageous economic situation and medical resources. Social and medical supports for maternal and infant health care are needed from outside. In addition, due to the fact that many local Yi people are undereducated and do not use mandarin [20], educational materials regarding HIV/AIDS should be tailored to use Yi language and be well understood by local people.

The prevalence of PTD was 5.8%. Consistent with existing research, maternal HIV infection was independently associated with PTD, with an OR of 1.8 in our study [39, 40]. Similar mechanisms may be shared between HIV infection with PTD and its association with LBW. Another important predictor of PTD was rural residency. A 2.1-fold increased risk of having PTD for pregnant women from rural area might result from less access to prenatal care and other medical resources compared with their counterparts from urban areas. In addition, pregnant women who deliver in county's MCH seems less likely to experience PTD as compared with women who deliver in people's hospital. It can be explained by the fact that MCHs in China are specialised hospitals in maternal and child care with better prenatal care resources, thus bearing lower prevalence of PTD.

Apgar score is an important indicator of newborn condition and prognosis [25], and a value of <7 is generally regarded as low and means medical attention is required for the infant. Our study found that infants born to HIV-positive mothers had a significantly higher prevalence of low Apgar score. This result is consistent with previous publications [3, 41]. Inadequate HIV infection detection and prenatal care are possible reasons. It is also generally recognised that people live in rural areas are less acceptable towards HIV/AIDS, which may result in less social support for HIV-infected mothers and thus affect pregnancy outcomes. Advanced maternal age (age ⩾35 years) also presented as an important factor that can influence pregnancy outcome, including low Apgar score of the newborn, with an increased odds of 1.7-fold, which is similar to the result from a previous study [42]. In addition, mothers who received C-section under medical indication were more likely to have a baby with a low Apgar score. This association is explainable since medically indicated C-section generally indicates health problems in the mother or signs of distress in the infant, which may lead the subsequent rating of newborn's Apgar scores to be low. During this process, medically indicated C-section can serve as a proxy for these underlying risk factors. Another important predictor for all three adverse pregnancy outcome indicators (LBW/PTD/low Apgar score) was pregnancy history. Women with two or more pregnancy histories surprisingly had less adverse pregnancy outcomes compared with primiparas. Future research is needed to explain such phenomenon.

Despite the above adverse pregnant outcomes, perinatal mortality is actually the worst pregnant outcome. Although it wasn't included as an outcome variable in our study, one of the most important goals for perinatal care is to reduce perinatal mortality among HIV-infected women. Nevertheless, the percentage of pregnant women using perinatal care services seemed to be extremely low. This suggests that active interventions are needed among pregnant women in this area. Possible interventions like training village doctors and public health workers in rural areas on health outreach work, including HIV counselling and testing, mobilising pregnant women, especially those from rural areas, to do early booking and HIV test at booking, and providing free medication and active medical services for HIV-infected women within local community.

To our knowledge, there is no large-scale study reporting HIV prevalence among maternal population in China and research on pregnancy outcomes of HIV-infected women are also limited. Our study filled this gap with hospital delivery data from a high-risk county of southwestern China. Findings of this study could provide data support for further investigation of pregnancy outcomes among HIV-positive population in China. In contrast to conventional methods, the multiple imputation we used enabled asymptotically unbiased estimation of the missing values and maximised use of available information from hospital deliver data. However, this study also suffers from several limitations. First, because of cross-sectional nature of the study, no causal conclusions can be drawn between potential influential factors and adverse pregnancy outcomes. Second, since most pregnant women did not utilise antenatal care services, thus not knowing their HIV status until hospital admission for delivery and were not on ART for this reason. As a result we were unable to obtain ART information, mothers’ antenatal care history and other information, and investigate their association with pregnancy outcomes. However, the impact of ART on pregnancy outcomes is believed to be small since most pregnant women did not start their treatment until admitted into hospital for delivery. Third, smoking and drug use during pregnancy are possible risk factor for adverse pregnancy outcomes, but we were unable to obtain such information from hospital delivery record [43, 44]. Nevertheless, the effect of maternal smoking on adverse pregnancy outcome, such as birthweight, is identified to be modified by maternal age, with increased effect on advanced maternal age [45, 46]. In our sample, majority (83.6%) of the maternal age were below 35 years of age. Thus, we believed the negative effect of maternal smoking on adverse pregnancy outcome is limited. As regard to drug use, it is a unique problem in this area, the role of drug use combined with HIV infection on pregnancy outcomes is worth investigating in future study. In terms of other confounding factors, our study does lack a comprehensive gathering of demographic information on mother-and-infant pair in this region, but we do hope this limited information we provided could benefit future research.

In conclusion, the prevalence of HIV infection was high among maternal population, suggesting a high prevalence of HIV infection among general population in this region. HIV-infected women had higher risks of experiencing adverse pregnancy outcomes, including LBW, PTD and low Apgar score. Rural residence predisposed adverse pregnancy outcomes. Advanced maternal age and being ethnic Yi have negative impact on rating of newborn's Apgar score. Findings of this study suggest more social and medical support for maternal-infant care needed for this region, especially in rural and Yi gathering place, selectively towards pregnant women with HIV infection and those with advanced age. Clinically, health outreach including HIV counselling and testing are needed in this region. Mobilise pregnant women to do early booking, HIV testing, and provide free medication and active medical services for HIV-infected women within the community are urgently needed. In addition, results of this study could provide data support for further investigation of pregnancy outcomes among HIV-positive population in China.

## Data

The dataset from this study is not publicly available. This is because they contain sensitive information which prohibited from distribution. But they are available from the corresponding author upon reasonable request and with permission from the original two hospitals. Interested researchers may contact School of public health, Fudan University, No.130 Dongan Rd., Xuhui District, Shanghai (200032), China. Phone: +86 21 54237974.
